# The PlaNet Consortium: A Network of European Plant Databases Connecting Plant Genome Data in an Integrated Biological Knowledge Resource

**DOI:** 10.1002/cfg.374

**Published:** 2004-03

**Authors:** H. Schoof, R. Ernst, K. F. X. Mayer

**Affiliations:** 1 Technische Universität München Chair of Genome-oriented Bioinformatics Center of Life and Food Science Freising-Weihenstephan D-85350 Germany; 2 Institute for Bioinformatics (MIPS) GSF National Research Centre for Environment and Health Ingolstäedter Landstraße 1 Neuherberg D-85764 Germany

## Abstract

The completion of the *Arabidopsis* genome and the large collections of other plant sequences generated in recent years have sparked extensive functional genomics efforts. However, the utilization of this data is inefficient, as data sources are distributed and heterogeneous and efforts at data integration are lagging behind. PlaNet aims to overcome the limitations of individual efforts as well as the limitations of heterogeneous, independent data collections. PlaNet is a distributed effort among European bioinformatics groups and plant molecular biologists to establish a comprehensive integrated database in a collaborative network. Objectives are the implementation of infrastructure and data sources to capture plant genomic information into a comprehensive, integrated platform. This will facilitate the systematic exploration of *Arabidopsis* and other plants. New methods for data exchange, database integration and access are being developed to create a highly integrated, federated data resource for research. The connection between the individual resources is realized with BioMOBY. BioMOBY provides an architecture for the discovery and distribution of biological data through web services. While knowledge is centralized, data is maintained at its primary source without a need for warehousing. To standardize nomenclature and data representation, ontologies and generic data models are defined in interaction with the relevant communities.Minimal data models should make it simple to allow broad integration, while inheritance allows detail and depth to be added to more complex data objects without losing integration. To allow expert annotation and keep databases curated, local and remote annotation interfaces are provided. Easy and direct access to all data is key to the project.


**Comparative and Functional Genomics**
*Comp Funct Genom* 2004; **5**: 184–189.
Published online in Wiley InterScience (www.interscience.wiley.com). **DOI:** 10.1002/cfg.374

**Conference Review**

### The PlaNet consortium: a network of European plant databases connecting plant genome data in an integrated biological knowledge resource

H. Schoof[1]*, R. Ernst[2] and K. F. X. Mayer[2]

[1] *Technische Universität München, Chair of Genome-oriented Bioinformatics, Centre of Life and Food Science, D-85350 Freising-Weihenstephan, Germany*

[2] *Institute for Bioinformatics (MIPS), GSF National Research Centre for Environment and Health, Ingolstäedter Landstraße 1, D-85764 Neuherberg, Germany*

**Correspondence to: H. Schoof, Technische Universität München, Chair of Genome-oriented Bioinformatics, Center of Life and Food Science, D-85350 Freising-Weihenstephan, Germany. E-mail: h.schoof@wzw.tum.de*

Received: 13 November 2003
Accepted: 24 November 2003

### Abstract

**The completion of the *Arabidopsis* genome and the large collections of other plant sequences generated in recent years have sparked extensive functional genomics efforts. However, the utilization of this data is inefficient, as data sources are distributed and heterogeneous and efforts at data integration are lagging behind. PlaNet aims to overcome the limitations of individual efforts as well as the limitations of heterogeneous, independent data collections. PlaNet is a distributed effort among European bioinformatics groups and plant molecular biologists to establish a comprehensive integrated database in a collaborative network. Objectives are the implementation of infrastructure and data sources to capture plant genomic information into a comprehensive, integrated platform. This will facilitate the systematic exploration of *Arabidopsis* and other plants. New methods for data exchange, database integration and access are being developed to create a highly integrated, federated data resource for research. The connection between the individual resources is realized with BioMOBY. BioMOBY provides an architecture for the discovery and distribution of biological data through web services. While knowledge is centralized, data is maintained at its primary source without a need for warehousing. To standardize nomenclature and data representation, ontologies and generic data models are defined in interaction with the relevant communities. Minimal data models should make it simple to allow broad integration, while inheritance allows detail and depth to be added to more complex data objects without losing integration. To allow expert annotation and keep databases curated, local and remote annotation interfaces are provided. Easy and direct access to all data is key to the project.**
**Copyright © 2004 John Wiley & Sons, Ltd.**

**Keywords:** **plant genome database; data integration; BioMOBY; annotation; ontology**

### Introduction

The future development of agricultural and environmental research relies strongly on plant gene data. Numerous functional genomics initiatives have started in the wake of the sequencing of the *Arabidopsis* genome, and large-scale sequence from other plant species is available, be it as genome or EST sequences [8,11]. However, complete genomes have only underlined the complexity of cellular life even more clearly: far from unravelling the blueprint of life, genomes are frequently

Copyright © 2004 John Wiley & Sons, Ltd.

seen as merely a parts list [12]. The need to understand gene function on all its levels, in all its interactions and in the context of regulatory processes has only become more obvious.

Many 'post-genomic' researchers are trying to address this issue, using methods such as the knock-out of every single gene in *Arabidopsis* using insertion mutagenesis, high-throughput two-hybrid protein interaction analysis or transcription analysis through whole-genome microarrays. These require extensive, highly integrated data sources for the evaluation of their results, e.g. for mapping co-expressed genes onto metabolic pathways while correlating them with common promoter elements. Current databases show several limitations (discussed in more detail in [6,7,16]):

- Online databases are distributed and data must be collected manually from multiple sources.
- Knowledge on what to find where is mostly not represented and must be gained by experience.
- Heterogeneous html output is difficult for humans to interpret and compare rapidly, and automatic processing is difficult.
- No integration between datasets requires finding related information through copy-and-paste into the search interfaces of each individual database.
- Heterogeneous data formats make data download and warehousing a tremendous effort.
- No common vocabularies or term definitions prevent data from being immediately comparable.

Thus, navigating and utilizing current databases is tedious and requires prior knowledge by the user. At the same time, the database curators face the challenge of ensuring timely updates, consistency, data richness, and expert annotation within their databases. These can frequently not be met, as generally, the resources to maintain a database are minimal. Michael Gribskov has stated four challenges for biological databases: integration, interoperation and federation; ontologies and defined semantics; community annotation and integration of analysis tools [6]. Whereas individual databases cannot keep up with these aims, several European plant genomics database providers got together to form the PlaNet project (**http://www.eu-plant-genome.net**) and address these issues.

### PlaNet: Aims and architecture

PlaNet is a distributed effort among bioinformatics groups and plant molecular biologists to establish a comprehensive integrated database in a collaborative network. This will help overcome the limitations of individual efforts as well as of the independent data collections. It creates a nucleus for other European and international groups and consortia to join and utilize the network. Overall objectives of the project are to:

- Capture genomic information into a comprehensive platform.
- Establish a network of dynamically interconnected European plant databases.
- Develop new methods for data exchange, database integration and access.
- Provide high quality integrated data resources for research.
- Ensure high availability of data generated by European laboratories and plant research consortia (data platform).
- Incorporate expert knowledge and regional networks.
- Focus direct contribution by regional plant research communities (expert annotation system).
- Perform systematic classification of plant genes and regulators.
- Develop standards for data representation and nomenclature.

PlaNet aims to provide a comprehensive plant genomics data platform allowing central access to integrated data from distributed sources. One approach towards data integration is warehousing, but this has severe drawbacks [16]: all data must be unified in a common database schema and all data regularly transformed and imported. However, as our knowledge increases, database schemas evolve to accommodate new data types or biological relationships, and it is extremely hard work to incorporate all these changes into the warehouse. A federated database allows the individual databases to continually work on their data representation, as long as they keep standardized interfaces intact that at least allow part of their data to be integrated. Where warehousing focuses on data translation, integration in a federated database emphasizes query translation [7].

Copyright © 2004 John Wiley & Sons, Ltd. 

Several additional advantages led the PlaNet project to opt for a federated approach. Know-how and experience on different aspects of genomics is available at different partner sites, leading these partners to create a specialized database. The federated approach allows know-how to remain in place, maintaining and curating the dataset that partner is specialized in, thus removing the risk that data, once imported into a warehouse, becomes stale as there is no expertise for curation at the site of the warehouse. Additionally, specialists can focus on a restricted, less complex dataset instead of having to deal with the complete warehouse. This facilitates rapid extensions and modifications of the specialized databases without necessarily involving the integration platform.

Another issue is the long-term sustainability of a warehouse. A federated database can remain functional, even if one or more of the partners no longer support it, but a centralized resource is usually doomed if the hosting partner can support it no longer. With respect to breadth, i.e. the broad coverage of relevant data sources, an important aspect is to keep the integration and connectivity layer lightweight, making it easy for data providers to integrate their database by implementing wrappers. This task is again best done locally, where detailed knowledge of the local schema is available.

The overall structure of the PlaNet federated database is shown in Figure 1. Data layer components are the specialized databases provided by the partners beside external public or community databases that are integrated through the partners. These data sources are interconnected to clients and between each other through a connectivity layer. Clients can be web interfaces for query and retrieval of data, or applications. These represent not only retrieval and analysis applications but also integration and consistency tools that perform data format transformations or updates and synchronization between the distributed databases.

### Implementation

The connectivity layer is realized with BioMoby (**http://www.biomoby.org**, [17]). BioMoby provides an architecture for the discovery and distribution of biological data through web services. A central registry contains data object definitions and service providers along with the data objects

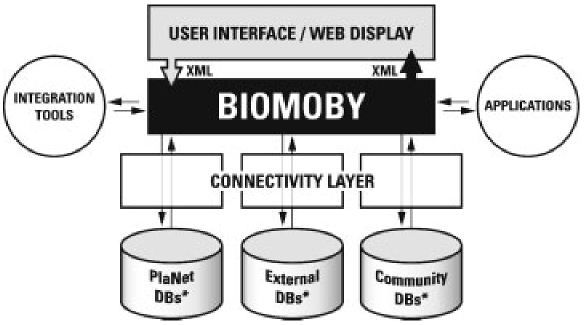

**Figure 1.** Architecture of the PlaNet federated database. The user interface and web display provides a single point of access to data and analysis tools provided by the distributed partners. To this end all databases are linked via a connectivity layer developed by PlaNet in collaboration with the BioMoby project [17]. The component databases make their data available as XML data objects through web services. Integration tools developed within PlaNet will be used to insure data consistency and cross-checking. Beside data sources, BioMoby also provides for the integration of analysis tools and applications the services act upon. Data objects are specified in the extensible mark-up language (XML) and are retrieved from web services using the simple object access protocol (SOAP). Essential components are ontologies for standardization of nomenclature and data models.

With respect to data objects, inheritance plays a vital role, allowing simple, basic objects to be defined that can easily be shared between multiple databases while allowing specialized, more complex objects to inherit from these. Specialized databases can represent all data relevant to them while maintaining exchangeability through the simpler parent object. Ontologies and standard data models to define the semantics of integrated data greatly facilitate integration [16]. The PlaNet project uses international standards where feasible, e.g. the gene ontology [1] or sequence ontology (**http://song.sourceforge.net**) but additional ontologies are created for areas not yet covered.

Once data sources are available through a common connectivity layer, client applications can be developed to perform integration tasks. This includes query interfaces, format transformations or data synchronization tasks. Query interfaces need to retrieve data from multiple sources. One approach, termed horizontal integration, is that any data type is mapped to a specific source for that type, i.e. retrieving different data from different

Copyright © 2004 John Wiley & Sons, Ltd. 

databases. Vertical integration is necessary when data of overlapping content is retrieved. For the purposes of PlaNet, both vertical and horizontal integration are encompassed, as several databases contain semantically equivalent data. This makes it necessary to handle inconsistent, contradictory or missing data. Tools to this end need to be developed, beside tools that allow closer integration by identifying synonyms or semantically equal entities in different databases, e.g. by checking sequence and additional attributes to identify identical genes, even if they are named differently.

A conclusion from the first stages of the project was that some effort must be invested into local database infrastructure in order to facilitate the implementation of wrappers that connect data sources to the connectivity layer. Multilayer architectures and separation of data model and presentation simplify this task and improve maintainability. A generic solution could be implemented by using BioRS, a commercial biological query and retrieval tool (**http://biors.gsf.de:8111**, **http://www.biomax.de**), to integrate local databases into a common query interface. This can be accessed through a CORBA-based proxy (DBProxy, **http://mips.gsf.de/proj/hnb/biors**). A generic BioMoby service that calls this proxy can then be used to instantiate a multitude of queries on the integrated data sources.

So far, the system has been tested with very simple objects, e.g. keywords, database identifiers like AGI locus codes or EMBL Accession Nos and simple sequences. Implemented services so far are retrieval services, e.g. retrieve sequence for EMBL Accession Nos or retrieve AGI locus codes for keywords. However, as Figure 2 demonstrates, new functionality can already be realized, utilizing data from distributed databases, e.g. through the linking of several services, starting from a keyword, a mutant phenotype can be retrieved from NASC (Nottingham *Arabidopsis* Stock Center: **http://arabidopsis.info**), in addition to the protein sequence from MAtDB (MIPS *Arabidopsis thaliana* Database [14]). Without the PlaNet connectivity layer, this requires two independent queries to the individual databases. As more services become available, this functionality will be extended, e.g. by adding a multiple sequence alignment service: the sequences retrieved by the keyword query could automatically be integrated into an alignment. Advanced client software could then colour code the sequences in the alignment by their functional classification, e.g. based on Gene Ontology [1] assignments.

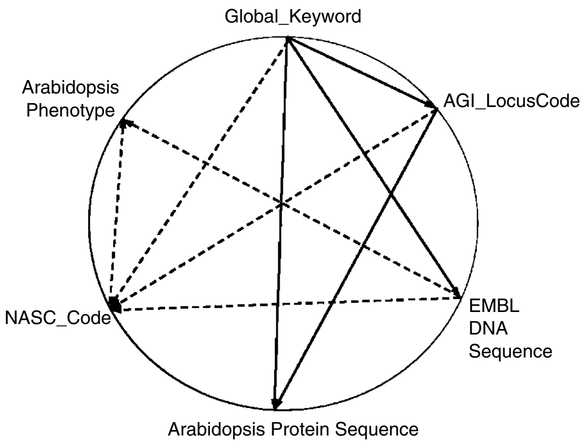

**Figure 2.** Services available through the PlaNet connectivity layer (not all shown here) are represented as arrows linking input and output data objects in this graph. As an example, starting from a keyword all corresponding AGI Locus Codes can be found. The AGI Locus Codes can then be used to retrieve the NASC Codes. With the NASC Code in hand another service can be queried to find the *Arabidopsis* phenotypes for them. This example workflow combines three services that operate on data from two different service providers (MIPS/Neuherberg and NASC/Nottingham). This workflow could also be automated in an application (see Figure 3). Complex queries can be realized through pipelining services and filters in a workflow where the output of one service is the input of another service. The implemented services are accessible through a simple, web-based query client prototype at **http://www.eu-plant-genome.net**

In order to test the usability of the BioMoby connectivity layer to automate integration in applications, a web application was built that gathers data from distributed sources via an embedded BioMoby client (Figure 3). This application queries the servers of all PlaNet partners for data and displays it. For the moment, this is basically a union of all retrieved data. However, this can be modified with little effort to perform more complex operations, such as removing redundancy to compile a superset of all data available (e.g. gather all *Arabidopsis* protein sequences and compile a non-redundant list).

Besides creating the infrastructure for a federated database, the second focus of PlaNet is to integrate and annotate plant genomics data. To this end, several partners set up specialized databases to

Copyright © 2004 John Wiley & Sons, Ltd. 

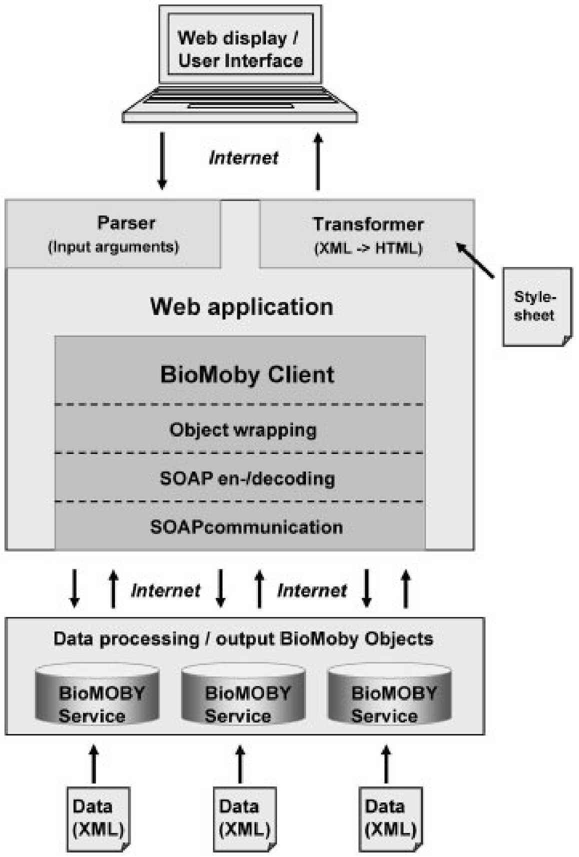

**Figure 3.** This figure demonstrates a simple prototype where the PlaNet connectivity layer is used for integration of distributed data in an application. A web application that displays details of the partner groups for the PlaNet homepage is shown. The data on each group (group members, research topics, address, etc.) are maintained by each group at their home server. The web application collects these data through calling BioMoby services at the distributed data sources, receiving XML data. This functionality is performed by an embedded BioMoby client that is reuseable for other purposes based on the query parameters passed to it. It wraps the data objects as required by the BioMoby specification, encodes a SOAP response and communicates with the BioMoby services at the remote partner sites. The returned XML data is unwrapped by the BioMoby client, then formatted as HTML through an XML stylesheet transformation and displayed represent functional genomics data from projects they are involved in. Automated pipelines to gather and integrate data are implemented, e.g. to align full-length cDNAs to the *Arabidopsis* genome or to map flanking sequence tags from insertion mutants to the genome [13]. This aims to improve and enhance the dataset available within PlaNet. In order to capture expert knowledge, annotation interfaces will be created and a user group of specialists established. Currently, tabular data on gene families is routinely integrated in MAtDB [13].

### Final remarks

For modern biology, a genome database that lists genes and their proposed functions is not enough. Results from functional genomics experiments, as well as bioinformatics analyses, need to be at the fingertips of the researcher in order to evaluate the genetic information in the context of biological pathways or cellular processes. An integrated data platform as provided by PlaNet will prove its value by the new questions that biologists will be able to ask it, and the new answers they will discover.

### Acknowledgements

PlaNet is funded by an EU Framework V grant; QLRI-CT-2001-00006.

### References

1. Ashburner M, Ball CA, Blake JA, *et al*. 2000. Gene ontology: tool for the unification of biology. The Gene Ontology Consortium. *Nature Genet* **25**: 25–29.
2. BIOMAX Informatics AG, distributor of BioRS: **http://www.biomax.de**
3. BioMoby: **http://biomoby.org**
4. BioRS online query form: **http://biors.gsf.de:8111/searchtool/searchtool.cgi**
5. DBProxy, a CORBA proxy for BioRS queries: **http://mips.gsf.de/proj/hnb/biors**
6. Gribskov M. 2003. Challenges in data management for functional genomics. *OMICS* **7**: 3–5.
7. Hernandez T, Kamphampati S. 2003. Integration of biological sources: current systems and challenges ahead. *ASU CSE TR-03–005*: **http://www.asu.edu**
8. Mayer KFX, Mewes HW. 2002. How can we deliver the large plant genomes? Strategies and perspectives. *Curr Opin Plant Biol* **5**(2): 173–177.
9. Nottingham *Arabidopsis* Stock Center (NASC): **http://arabidopsis.info**
10. PlaNet project: **http://www.eu-plant-genome.net**
11. Rudd S, Mewes HW, Mayer KFX. 2003. Sputnik: a database platform for comparative plant genomics. *Nucleic Acids Res* **31**: 128–132.
12. Schoof H. 2003. Towards interoperability in genome databases: the MAtDB (MIPS *Arabidopsis thaliana* database) experience. *Comp Funct Genom* **4**: 255–258.
13. Schoof H, Ernst R, Nazarov V, *et al*. 2004. MIPS *Arabidopsis thaliana* Database (MAtDB): an integrated biological

Copyright © 2004 John Wiley & Sons, Ltd. 

knowledge resource for plant genomics. *Nucleic Acids Res* **32**: D373–D376.
14. Schoof H, Zaccaria P, Gundlach H, *et al.* 2002. MIPS *Arabidopsis thaliana* database (MAtDB): an integrated biological knowledge resource based on the first complete plant genome. *Nucleic Acids Res* **30**(1): 91–93.
15. Sequence ontology: **http://song.sourceforge.net**
16. Stein L. 2003. Integrating biological databases. *Nature Rev Genet* **4**: 337–345.
17. Wilkinson MD, Links M. 2002. BioMOBY: An open source biological web services proposal. *Briefings Bioinformat* **3**: 331–341.

Copyright © 2004 John Wiley & Sons, Ltd. 
